# The multidimensional assessment of interoceptive awareness, version 2: Translation and psychometric properties of the Chinese version

**DOI:** 10.3389/fpsyt.2022.970982

**Published:** 2022-11-11

**Authors:** Binyu Teng, Dan Wang, Conghui Su, Hui Zhou, Tengfei Wang, Wolf E. Mehling, Yuzheng Hu

**Affiliations:** ^1^Department of Psychology and Behavioral Sciences, Zhejiang University, Hangzhou, China; ^2^National Clinical Research Center for Child Health, The Children’s Hospital, Zhejiang University School of Medicine, Hangzhou, China; ^3^Department of Family and Community Medicine, University of California, San Francisco, San Francisco, CA, United States; ^4^Osher Center for Integrative Medicine, University of California, San Francisco, San Francisco, CA, United States

**Keywords:** multidimensional assessment of interoceptive awareness, reliability, validity, interoception, interoceptive awareness, MAIA-2

## Abstract

**Background:**

The Multidimensional Assessment of Interoceptive Awareness (MAIA) is a self-report questionnaire developed by Dr. Mehling that has been widely used to assess multiple dimensions of interoceptive awareness. To further improve the MAIA, Mehling developed the Multidimensional Assessment of Interoceptive Awareness, Version 2 (MAIA-2). The goal of this study is to systematically translate the MAIA-2 into Chinese and to investigate the psychometric properties of the Chinese version (MAIA-2C).

**Materials and methods:**

The translation and adaptation of the questionnaire was conducted according to Beaton’s method. A total number of 627 participants were enrolled and completed the survey. The entire sample was randomly divided into a training sample (*n* = 300, 47.8%) and a validation sample (*n* = 327, 52.2%) for a cross-validation. Exploratory factor analysis (EFA) was used to identify the factor structure of the MAIA-2C in the training sample while confirmatory factor analysis (CFA) was used to test the factor structure obtained by EFA. The reliability of the MAIA-2C was indicated by Cronbach’s alpha. The convergent and discriminant validity were assessed by Pearson intercorrelations between the MAIA-2C and the Five-Facet Mindfulness Questionnaire (FFMQ) and State-Trait Anxiety Inventory-Trait anxiety (STAI-T).

**Results:**

The EFA results showed an initial 10-factor model, but some items (1, 2, 3, 4, 15, and 16) were deleted because they did not yield the original subscale construct, eventually resulting in a 7-factor model. The CFA results represented a good model fit (χ^2^/df = 2.170, RMSEA = 0.060, SRMR = 0.0810, CFI = 0.890). The Cronbach’s alpha was 0.822 for the total scale and ranged from 0.656 to 0.838 for the subscales. The results of convergent and discriminant validity showed that most MAIA-2C subscales were correlated with the average score and subscales of FFMQ (*r* = −0.342∼0.535, *p* < 0.05), and all of the subscales of the MAIA-2C showed negative correlations with the STAI-T total score (*r* = −0.352∼−0.080, *p* < 0.05).

**Conclusion:**

The MAIA-2C is a valid and reliable instrument for evaluating multiple dimensions of interoceptive awareness in a Chinese population.

## Introduction

Interoception is defined as the perception of internal bodily changes (1). Recently, a revised description of interoception has been proposed with more details, including the processes by which an organism senses, interprets, integrates, and regulates signals from inside the body. This revision expands the communication from the brain to other physiological systems through descending pathways (2).

As a multidimensional construct, interoception consists of three psychological dimensions: interoceptive awareness (sensibility, referring to one’s beliefs and consciousness about their interoceptive ability), accuracy (sensitivity, the performance and reliability with objective tests of internal detection, such as heartbeats), and the metacognitive dimension (accurate perception on one’s own interoceptive performance) (1, 3). Many studies have demonstrated the role for interoception in cognitive functioning such as decision-making, memory, and emotion processing. In addition, it has been found that the interoceptive function decreased with age, and the decline accounted for some aspects of cognitive impairment and age-related health issues (4).

Interoceptive accuracy is thought to play an important role in interoception (5). Many previous studies have found that interoceptive awareness might be influenced by many trait-like characteristics such as trait anxiety and self-esteem (6). Interoceptive awareness has been proposed to mediate the health benefits of mind-body interventions in daily life. These interventions include Qigong, Taichi, yoga, mindfulness, and others (7–9). Researchers have long been interested in the psychological mechanisms of mind-body interventions. And so, it is necessary to assess these mechanisms—interoceptive awareness has been suggested as one of them (10).

To evaluate the effectiveness of mind-body interventions, it is crucial to have validated instruments to assess interoceptive awareness (7, 11). The Body Perception Questionnaire (12) was one of the most commonly used questionnaires. However, the BPQ is a unidimensional biological measure of one’s interoceptive awareness of anxiety-related sensations (13), which is often used in the biological studies (14). Different with BPQ, Dr. Mehling (8) developed a self-report questionnaire, the Multidimensional Assessment of Interoceptive Awareness (MAIA). It can assess the most salient facets and capture any changes in multiple dimensions of interoceptive awareness. The MAIA comprises 32 items and 8 distinct subscales. The eight subscales are defined as Noticing, Not-Distracting, Not-Worrying, Attention Regulation, Emotional Awareness, Self-Regulation, Body Listening, and Trusting (8). The MAIA has become one of the most widely used self-report measures of interoceptive awareness. To date, it has been translated into 26 languages, and 12 of these versions have been validated with good reliability (15).

To further improve the MAIA, Mehling (16) developed the Multidimensional Assessment of Interoceptive Awareness, Version 2 (MAIA-2). Compared to the original version, MAIA-2 retains the eight subscales but consists of 37 items. First, in the Not-Distracting scale, there are three new items: (1) *I try to ignore pain* (R); (2) *I push feelings of discomfort away by focusing on something else* (R), and (3) *When I feel unpleasant body sensations, I occupy myself with something else so I don’t have to feel them* (R). Second, in the Not-Worrying scale, the two new items are: (1) *I can stay calm and not worry when I have feelings of discomfort or pain* and (2) *When I am in discomfort or pain, I can’t get it out of my mind* (R). R indicates reverse scoring. The Cronbach alphas of the two scales were improved (Not-Distracting: 0.74; Not-Worrying: 0.67).

In order to apply the MAIA-2 to the Chinese population, this study was conducted to systematically translate it into Chinese and to investigate the psychometric properties of the Chinese version of the MAIA-2 (MAIA-2C).

## Materials and methods

### Participants

In the survey stage, we recruited 853 young adults from Zhejiang University. During the survey, a catch-trial was set among the items for quality control by asking participants to choose one specified option. We also examined the filling time and excluded participants whose response time was below 200 s (*n* = 121) or above 1,200 s (*n* = 45). The final sample of the present study consisted of 627 participants aged between 18 and 26 years (*M* = 21.62, *SD* = 2.44, 38.3% male and 61.7% female). All the participants were native Chinese. This study was approved by the Ethics Committee. Participants received a financial reward (3 RMB) for their participation.

### Instruments

In accordance with the original MAIA study (8), the following questionnaires (FFMQ and STAI-T) were used to test the psychometric properties of the MAIA-2C.

#### Multidimensional assessment of interoceptive awareness, version 2

To measure multiple dimensions of interoception bodily awareness, the original MAIA consists of 32 items with 8 subscales (8). Based on the original version, MAIA-2 still retains an 8-factor structure (16). These are (i) Noticing: the awareness of uncomfortable, comfortable, and neutral body sensations; (ii) Not-Distracting: the tendency not to ignore or distract oneself from sensations of pain or discomfort; (iii) Not-Worrying: the tendency not to experience emotional distress or worry with sensations of pain or discomfort; (iv) Attention Regulation: the ability to sustain and control attention to body sensations; (v) Emotional Awareness: the awareness of the connection between body sensations and emotional states; (vi) Self-Regulation: the ability to regulate psychological distress by attention to body sensations; (vii) Body Listening: actively listening to the body for insight, and (viii) Trusting: the experiences of one’s body as safe and trustworthy. However, in contrast to MAIA, the items of MAIA-2 were increased to 37. In the MAIA-2, the items are tested on a 6-point Likert scale (0–5), taking the average rating of all the items on each scale as the score, with higher scores indicating a higher ability of interoceptive bodily awareness. The MAIA-2 subscale Cronbach’s alphas ranged from 0.64 to 0.83 (16).

#### Five-facet mindfulness questionnaire

The Chinese version of the FFMQ (17, 18) was selected as a measure to assess the convergent and discriminant validity of the MAIA-2C. The FFMQ is a 39-item self-report instrument with five subscales: (1) Observing: the ability to notice internal stimuli among other stimuli, such as body sensations, emotion, and others; (2) Describing: the ability to note or describe internal experience; (3) Acting with Awareness: attending to one’s current activities; (4) Non-judging of Inner Experience: evaluating one’s body sensations; and (5) Non-reactivity to Inner Experience: accepting thoughts and feelings without being absorbed in them. Items are answered on a five-point Likert scale (1–5). Internal-consistency reliabilities in the FFMQ subscales were between 0.75 and 0.91 (17), and Chinese reliabilities ranged from 0.45 to 0.84 (18).

#### State-trait anxiety inventory

The Chinese version of the STAI was validated by Li and Qian (19), and the STAI-T subscale was also used to assess the convergent and discriminant validity of the MAIA-2C. The STAI-T subscale is a 20-item self-report questionnaire with a four-point Likert rating from 1 (almost never) to 4 (almost always) (20). Both FFMQ and STAI-T were used for convergent and discriminant validation as in previous work (8).

### Procedure

The translation and adaptation of the questionnaire was conducted by Beaton’s method (21) as follows:

#### Forward-backward translation

After obtaining permission from the original author, Dr. Wolf Mehling, to translate the MAIA-2 a forward-backward translation of the English MAIA-2 into Chinese was conducted to retain invariance meaning across different cultures (21). The forward-backward translation process includes the following steps:

• Three native Chinese bilingual speakers, two did not know the construct and one was familiar with the construct, completed the forward-translation into Chinese independently.
• After comparing the three translated versions, we discussed them with one native Chinese bilingual professor and formed a forward-translated version.
• A bilingual overseas doctoral student, who was not familiar with the construct and blinded to the original English version, finished the back-translation into English according to the forward-translated document.
• After comparing the back-translation and the original English version, divergences were identified and discussed with the original author of the MAIA-2. The Chinese version was modified accordingly, and the translation process was completed. The cognitive interviews and survey studies were then conducted (see below).

#### Cognitive interviews

A total of 8 interviewees (2 males, 6 females) aged between 19 and 54 years (*M* = 30.75, *SD* = 12.18) participated in the cognitive interviews. Participants received financial compensation (30 RMB) for their participation.

At this stage, interviewees were asked to complete the translated questionnaire and note any questions or doubts they had about the items. For example, if they did not understand the item or if there was ambiguity in the item. After they had completed the questionnaire, we started to conduct the cognitive interviews by asking them in-depth questions that they wrote down. Then, we randomly selected some items and asked interviewees to elaborate their meanings. Finally, we sorted all the questions and divergences and discussed them with the original author of the MAIA-2. Some modifications were made, and a final translated version was formed.

#### Survey

The translated MAIA-2 was self-administered using a web platform.^1^ Before filling out the questionnaires, the purpose of the research was explained to the participants, and the consent information was presented. The participants could only proceed after agreeing to the consent. They were asked to complete the Likert scales as well as demographic characteristics including age and gender. Participants received financial compensation (3 RMB) for participation.

### Data analysis

To evaluate the factor structure of the scale, a cross-validation procedure was completed in a total sample of 627, which was randomly divided into a training sample (*n* = 300, 47.8%) and a validation sample (*n* = 327, 52.2%).

The training sample was used for an exploratory factor analysis (EFA) to identify the factor construct of the MAIA-2C. The EFA was performed with a maximum-likelihood estimation and varimax rotation (extraction criterion: eigenvalue > 1).

The validation sample was used for a confirmatory factor analysis (CFA) to test the factor construct obtained with the EFA. Parameters were estimated using the maximum-likelihood estimation method. The fit statistics were evaluated based on the criteria recommended by Kline (22) and DiStefano (23). Specifically, the model fit was considered good (or acceptable) if normed χ^2^ (= χ^2^/df) ≤ 2 (3), RMSEA ≤ 0.06 (0.08), SRMR ≤ 0.08 (0.10), and CFI ≥ 0.95 (0.90).

Cronbach’s alpha coefficient was used to evaluate the reliability of the scale and the subscales. If the Cronbach’s alpha of the scale > 0.7, it was considered acceptable. To examine associations between items and relationships between subscales, the Pearson correlation matrix was used. The convergent and discriminant validity of the MAIA-2C were assessed by Pearson intercorrelations between the MAIA-2C and FFMQ and STAI-T.

Statistical analyses were conducted using *IBM*^®^ *SPSS Statistics 26* and *IBM*^®^ *SPSS AMOS 23*.

## Results

### Translation of multidimensional assessment of interoceptive awareness Chinese version

We used a sample consisted of 627 participants recruited from Zhejiang University. The adaptation was formed using a forward-backward translation. Cognitive interviews brought us to understand the essence of most items, rendering the translation more culturally adapted. We identified difficulties in comprehension for Items 5, 10, 12, 18, 21, 35, and 37 and discussed biases and ambiguities with Dr. Mehling, the author of the original MAIA. Then some adaptations were made. Some important biases were below: For Item 12, some interviewees did not understand “what’s wrong” with my body or life (wrong with my body). For Item 35, it was difficult to understand “feel at home” in Chinese (Being at home in the body implies a sense of comfort and trust).

### Univariate descriptive statistics for the items

A Kaiser–Meyer–Olkin (KMO) sampling adequacy of 0.821 and a significant Bartlett test of sphericity (χ^2^ = 4454.66; *p* < 0.001) showed an appropriate model for analyzing the data. Assessment of skewness and kurtosis showed that most item scores ranged from -1 to 1, which could infer an approximation of each item to a normal distribution (Supplementary Table 1). The kurtosis of items 2 (1.448), 11 (1.065), and 37 (1.127) were out of the range between −1 and 1, but they were also close to 1 and below 1.5, which was considered acceptable. Given that each item has six possible response choices, we used the ML method to estimate the model parameters. This method showed robustness when each item of a scale had an approximately normal distribution (24, 25).

### Results from exploratory factor analysis

The EFA was conducted with maximum-likelihood estimation and varimax rotation (extraction criterion: eigenvalue > 1) (Supplementary Table 2). The results showed a 10-factor model. But only Item 15 belonged to Factor 10, and so Item 15 was removed. Items 1–4 originally belonging to Noticing were distributed into two independent subscales, which had not met a minimal threshold number of subscale item. Also, the Cronbach’s alpha of the Noticing subscale, including Items 1–4 (0.582), was below 0.6 in our sample. Given the above results, we removed these items. In addition, Item 16 was removed because it did not distribute to the subscale to which it theoretically belonged. The Not-Worrying subscale had a relatively low reliability 0.638, but it was close to 0.7, and so the scale was retained. Thus, our EFA reduced the MAIA-2C from 37 to 31 items, with a seven-factor model (Supplementary Table 3).

The commonalities reproduced by the varimax rotation ranged between 0.36 and 0.79, and the seven extracted factors explained 61.2% of the total variance.

### Results from confirmatory factor analysis

**FIGURE 1:**
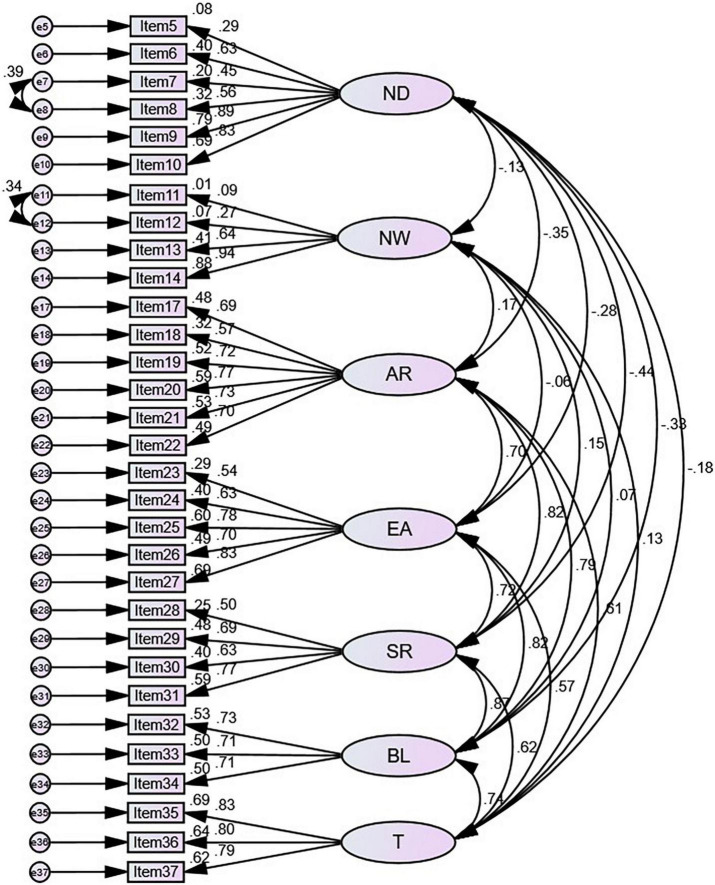
Structural model of adaptation to the multidimensional assessment of interoceptive awareness Chinese version (MAIA-2C). N, Noticing; ND, Not-Distracting; NW, Not-Worrying; A, Attention Regulation; E, Emotional Awareness; S, Self-Regulation; B, Body Listening; T, Trusting.

After conducting the CFA, the goodness of the fit statistics of the 7-factor model were normed χ^2^ (χ^2^/*df*) = 2.375 ≤ 3, a RMSEA = 0.065 ≤ 0.08, a SRMR = 0.0829 ≤ 0.1, a CFI = 0.870 ≤ 0.9. Given the similarities of items in the same subscale, we made a correlation between the residuals of Item 7 (When I feel pain or discomfort, I try to power through it.) and Item 8 (I try to ignore pain.), because they both contribute to the subscale Not-Distracting and focus on how to deal with pain. And we also made a correlation between the residuals of Item 11 (When I feel physical pain, I become upset.) and Item 12 (I start to worry that something is wrong if I feel any discomfort.), because they both contribute to the subscale Not-Worrying and focus on the state of “upset and be worried.” After making two correlations above, the goodness of the fit statistics of the model (Figure 1) were normed χ^2^ (χ^2^/*df*) = 2.170 ≤ 3, a RMSEA = 0.060 ≤ 0.08, a SRMR = 0.0810 ≤ 0.1, a CFI = 0.890 ≤ 0.9.

### Reliability of multidimensional assessment of interoceptive awareness Chinese version

The Cronbach’s alpha of the MAIA-2C was 0.822, and subscales ranged from 0.656 to 0.838. The subscale-subscale correlation analysis indicates that the Not-Distracting scale has an inverse correlation with Attention Regulation (*r* = −0.252, *p* < 0.01), Emotional Awareness (*r* = −0.102, *p* < 0.05), Self-Regulation (*r* = −0.233, *p* < 0.01), Body Listening (*r* = −0.143, *p* < 0.01), and Trusting (*r* = −0.105, *p* < 0.01), and does not show a significant correlation with Not-Worrying. Also, the Not-Worrying scale has an inverse correlation with Emotional Awareness (*r* = −0.237, *p* < 0.01) and Body Listening (*r* = −0.103, *p* < 0.01), and does not show any significant correlations with the other subscales. Correlations between each subscale were presented in Table 1.

**TABLE 1:** Pearson product-moment correlations among the seven multidimensional assessment of interoceptive awareness (MAIA) scales and Cronbach’s alpha.

	ND	NW	AR	EA	SR	BL	T
Not-distracting	0.803						
Not-worrying	–0.055	0.656					
Attention regulation	−0.252**	–0.032	0.822				
Emotional awareness	−0.102*	−0.237**	0.524**	0.817			
Self-regulation	−0.233**	–0.017	0.546**	0.463**	0.741		
Body listening	−0.143**	−0.103**	0.563**	0.581**	0.571**	0.765	
Trusting	−0.105**	–0.035	0.430**	0.419**	0.446**	0.532**	0.838

Cronbach’s alpha on the diagonal. *p < 0.05, **p < 0.01.

### Validity of multidimensional assessment of interoceptive awareness Chinese version

Convergent and discriminant validity was analyzed by calculating the Pearson correlations of the adapted MAIA-2C scales (7 factors) and the scores of FFMQ and STAI-T (Table 2). As shown in Table 2, most of the MAIA-2C scales are significantly and positively correlated with the scores of the FFMQ subscales and the total FFMQ score, but the Not-Worrying subscale does not show significant correlations with subscale Describing in FFMQ and the total score. Further, the Acting With Awareness scale belonging to FFMQ does not show any significant correlations with the Attention Regulation, Self-Regulation, and Trusting subscales in the MAIA-2C. There were also some significant negative correlations between some dimensions of both scales, such as the Non-judging of Inner Experience subscale in FFMQ with the Attention Regulation, Emotion Awareness, Self-Regulation, Body Listening, and Trusting subscales in the MAIA-2C. Regarding the STAI-T, all of the subscales of the MAIA-2C showed significant correlations with the STAI-T total score.

**TABLE 2:** Pearson’s correlations of the five-facet mindfulness questionnaire (FFMQ), state-trait anxiety inventory-trait anxiety (STAI-T), and multidimensional assessment of interoceptive awareness Chinese version (MAIA-2C).

	ND	NW	AR	EA	SR	BL	T
**FFMQ**							
OBS	−0.107**	−0.163**	0.443**	0.513**	0.399**	0.535**	0.353**
DSC	0.083*	0.016	0.311**	0.215**	0.189**	0.260**	0.299**
AWA	0.236**	0.093*	–0.044	−0.123**	–0.032	−0.136**	0.043
NOJ	0.252**	0.170**	−0.282**	−0.293**	−0.239**	−0.342**	−0.143**
NOR	−0.220**	0.087*	0.468**	0.328**	0.463**	0.397**	0.269**
Total	0.127**	0.073	0.332**	0.238**	0.283**	0.262**	0.321**
**STAI-T**							
Total	−0.106**	−0.141**	−0.193**	−0.080*	−0.257**	−0.107**	−0.352**

MAIA-2C: N, Noticing; ND, Not-Distracting; NW, Not-Worrying; A, Attention Regulation; E, Emotional Awareness; S, Self-Regulation; B, Body Listening; T, Trusting. FFMQ: OBS, Observing; DSC, Describing; AWA, Acting with Awareness; NOJ, Non-judging of Inner Experience; NOR, Non-reactivity to Inner Experience. *p < 0.05, **p < 0.01.

## Discussion

The MAIA-2 was systematically translated into Chinese and validated in young adults with good psychometric properties.

We used the EFA to obtain a seven-factor model. Although the results showed a 10-factor model, Items 15 and 16 did not belong to the factors that they theoretically belong to, and the original MAIA scale “Noticing” was deleted from the MAIA-2C because the Cronbach’s alpha coefficient of this scale (0.582) was below 0.6. A new rotated factorial matrix was established for the 31-item scale.

The low contribution of Item 15 (When I am in discomfort or pain, I can’t get it out of my mind.) to the subscale Not-Worrying (the result showed it belonged to a new independent factor) might be due to the order in which Items 13 and 14 are positively scored, whereas Item 15 is reversely scored. The content of Item 15 focuses on the ability “get the discomfort and pain out of mind” whereas other items focus on the state “upset and being worried.” The low contribution of Item 16 (I can pay attention to my breath without being distracted by things happening around me.) to the subscale Attention Regulation (the result showed it belonged to Self-Regulation) might be due to the focus on “breath” while other items emphasize “body.” Items 30 and 31 from the subscale Self-Regulation also concentrate on “breath.” Therefore, after conducting EFA, Item 16 was classified as Self-Regulation with Items 30 and 31.

As for Cronbach’s alpha, six of the seven subscales were above 0.7, which showed a good internal consistency. The reliability of the subscales Not-Worrying (0.656) was questionable, but it was very close to the original version of the MAIA-2 (0.67) (16). One possible interpretation is that it is the only dimension that has both positive and negative scorings, and reliability is influenced by the number of items in the subscale, which usually increases with the number (25). Therefore, removing Item 15 might weaken its reliability.

The CFA was conducted to show the goodness of fit statistics of seven-subscale model. One limitation of the present study was that CFI (0.890) of the model fit was less than 0.900, indicating a slightly poor model fit. It might be due to the fact that some items were correlated at a relatively high level. For example, when allowing the correlation between two items regarding listening (i.e., Item 33 and 34 in the model), the CFI would exceed 0.9.

For the convergent construct validity, most of the seven subscales were significantly and positively correlated with the scores of the FFMQ subscales and the total FFMQ score. FFMQ is widely used to assess the nature of mindfulness, and interoceptive awareness is thought to be one of the psychological mechanisms of mind-body interventions. We obtained a similar survey result to that of the original English MAIA study (8) and a study of the Japanese version (26). This showed that the MAIA-2C is a useful measurement of mindful bodily awareness. However, in contrast to the original English MAIA, our results showed that the Not-Worrying subscale does not show significant correlations with the subscale Describing in FFMQ, and the total score and the Acting With Awareness scale belonging to FFMQ do not show any significant correlations with Attention Regulation, Self-Regulation, and Trusting subscales in the MAIA-2C. There were also some significant negative correlations between some dimensions of both scales, such as the Non-judging of Inner Experience subscale in FFMQ with Attention Regulation, Emotion Awareness, Self-Regulation, Body Listening, and Trusting subscales in the MAIA-2C. However, in the original MAIA study, all of the MAIA subscales were significantly positively correlated with the FFMQ subscales (8). One possible reason for this is the difference in the sample population. The original sample was from participants experienced with mind-body practices, while our sample was from young adults who have fewer mind-intervention experiences. Our result was equivalent to the Japanese study (26), whose individuals in the sample also had fewer experiences with mind-body practices.

Regarding the STAI-T, all of the subscales of the MAIA-2C were significantly negatively correlated with the STAI-T total score (-0.080 to -0.352). This suggests that trait anxiety is also negatively associated with bodily awareness measures of the MAIA-2C. These results were also consistent with the original MAIA study (8) and the Japanese-version study (26), demonstrating all negative correlations between the MAIA and STAI-T scores.

The first version of MAIA had been translated into Chinese by Lin et al. (7), using the same translation procedure as we did. Except some inherent differences between the two original versions, there are several potential factors may differentiate Lin’s version and ours. First, the MAIA-C was expressed in traditional Chinese while ours in simplified Chinese. There are some subtle differences in the favor of wording when describing the same thing. Therefore, our version is more appropriate when participants are from mainland of China. Second, as language is the media of custom and culture, there would be some differences between the MAIA and MAIA-2C as the validations were based upon a China Taiwan population and a China mainland population, respectively.

In summary, the translated and verified MAIA-2C has reasonable reliabilities and validities. For further study, different Chinese samples should be investigated. As noted above, some participants with more experiences in mind-body practices could be used to test the reliability and validity of MAIA-2C.

## Conclusion

Our study provides a measurement of interoceptive awareness adapted to a China sample. The MAIA-2C is a reliable and valid instrument for evaluating multiple dimensions of interoceptive awareness in a Chinese population—an instrument that could be used for future research.

## Data availability statement

The raw data supporting the conclusions of this article will be made available by the authors, without undue reservation.

## Ethics statement

The studies involving human participants were reviewed and approved by Children’s Hospital of Zhejiang University. The patients/participants provided their written informed consent to participate in this study.

## Author contributions

BT: conceptualization, investigation, data curation, formal analysis, and writing—original draft. DW: data curation and writing—review and editing. CS and HZ: data curation. TW: data curation and formal analysis. WM: original author and writing—review. YH: conceptualization, data curation, writing—review and editing, and funding acquisition. All authors contributed to the article and approved the submitted version.

## Footnotes

1
www.wjx.cn
