# Supplementary material for: The multidimensional assessment of interoceptive awareness, version 2: Translation and psychometric properties of the Chinese version
Source: Front Psychiatry. 2022 Nov 11;13:970982. doi: 10.3389/fpsyt.2022.970982 (PMC9691670; doi:10.3389/fpsyt.2022.970982)
Supplement: Supplementary file 1 [file Data_Sheet_1.docx]

Supplementary Material

**Supplementary eTable 1.**

**Univariate descriptive statistics for the items (*n* = 300).**

| Item | Mean | Confidence interval (95%) | | Variance | Skewness | Kurtosis |
| --- | --- | --- | --- | --- | --- | --- |
| 1 | 3.003 | 2.863 | 3.133 | 1.212 | -0.654 | -0.068 |
| 2 | 3.750 | 3.650 | 3.847 | 0.858 | -0.805 | 1.448 |
| 3 | 3.117 | 2.970 | 3.267 | 1.242 | -0.307 | -0.576 |
| 4 | 3.283 | 3.150 | 3.437 | 1.209 | -0.410 | -0.498 |
| 5 | 2.943 | 2.813 | 3.083 | 1.213 | -0.174 | -0.548 |
| 6 | 2.120 | 1.983 | 2.263 | 1.240 | 0.300 | -0.595 |
| 7 | 1.483 | 1.370 | 1.620 | 1.074 | 0.785 | 0.520 |
| 8 | 2.240 | 2.097 | 2.387 | 1.268 | 0.165 | -0.708 |
| 9 | 1.797 | 1.680 | 1.930 | 1.128 | 0.492 | -0.224 |
| 10 | 2.023 | 1.897 | 2.153 | 1.152 | 0.192 | -0.652 |
| 11 | 1.177 | 1.057 | 1.307 | 1.056 | 0.961 | 1.065 |
| 12 | 1.717 | 1.583 | 1.857 | 1.273 | 0.447 | -0.560 |
| 13 | 2.063 | 1.937 | 2.200 | 1.151 | 0.194 | -0.450 |
| 14 | 2.240 | 2.107 | 2.383 | 1.209 | 0.251 | -0.427 |
| 15 | 2.143 | 2.020 | 2.257 | 1.074 | 0.233 | -0.272 |
| 16 | 2.120 | 1.993 | 2.250 | 1.094 | 0.177 | -0.403 |
| 17 | 2.740 | 2.613 | 2.870 | 1.127 | -0.039 | -0.315 |
| 18 | 3.077 | 2.957 | 3.207 | 1.114 | -0.284 | -0.428 |
| 19 | 2.840 | 2.723 | 2.953 | 1.025 | -0.050 | -0.365 |
| 20 | 2.660 | 2.537 | 2.783 | 1.075 | 0.174 | -0.402 |
| 21 | 2.747 | 2.633 | 2.870 | 1.104 | -0.085 | -0.297 |
| 22 | 2.797 | 2.667 | 2.930 | 1.116 | -0.216 | -0.307 |
| 23 | 2.883 | 2.747 | 3.020 | 1.186 | -0.196 | -0.514 |
| 24 | 3.197 | 3.073 | 3.313 | 1.050 | -0.593 | 0.221 |
| 25 | 3.253 | 3.123 | 3.377 | 1.125 | -0.540 | 0.038 |
| 26 | 3.207 | 3.067 | 3.350 | 1.263 | -0.425 | -0.408 |
| 27 | 3.177 | 3.037 | 3.310 | 1.179 | -0.224 | -0.671 |
| 28 | 2.023 | 1.887 | 2.153 | 1.155 | 0.518 | -0.124 |
| 29 | 2.913 | 2.780 | 3.060 | 1.156 | -0.287 | -0.346 |
| 30 | 2.873 | 2.730 | 3.007 | 1.192 | -0.218 | -0.423 |
| 31 | 2.617 | 2.483 | 2.753 | 1.212 | -0.048 | -0.556 |
| 32 | 3.170 | 3.043 | 3.303 | 1.082 | -0.502 | -0.061 |
| 33 | 2.373 | 2.233 | 2.520 | 1.235 | 0.203 | -0.654 |
| 34 | 2.603 | 2.463 | 2.733 | 1.162 | 0.018 | -0.464 |
| 35 | 2.920 | 2.790 | 3.053 | 1.124 | -0.168 | -0.429 |
| 36 | 3.090 | 2.960 | 3.227 | 1.197 | -0.351 | -0.428 |
| 37 | 3.467 | 3.357 | 3.570 | 0.955 | -0.774 | 1.127 |

**Supplementary eTable 2.**

**Items and Exploratory Factor Analysis (EFA) loadings of the Chinese version of the MAIA-2.**

| Item | Factors | | | | | | | | | | Original Scale | Chinese Version |
| --- | --- | --- | --- | --- | --- | --- | --- | --- | --- | --- | --- | --- |
|  | 1 | 2 | 3 | 4 | 5 | 6 | 7 | 8 | 9 | 10 |  |  |
| 1 | -0.06 | 0.24 | 0.13 | -0.04 | -0.15 | -0.07 | 0.02 | 0.19 | **0.68** | -0.12 | N | N-1 |
| 2 | -0.10 | 0.15 | 0.13 | -0.04 | 0.14 | -0.12 | 0.03 | **0.66** | 0.18 | -0.04 | N | N-2 |
| 3 | -0.08 | 0.06 | 0.19 | 0.17 | 0.19 | -0.07 | 0.11 | 0.07 | **0.68** | 0.01 | N | N-1 |
| 4 | -0.05 | 0.10 | 0.07 | 0.24 | 0.02 | 0.08 | 0.23 | **0.52** | 0.19 | -0.13 | N | N-2 |
| 5 | **0.46** | 0.20 | -0.16 | 0.02 | 0.21 | -0.06 | -0.16 | 0.23 | -0.16 | 0.36 | ND | ND |
| 6 | **0.71** | 0.05 | -0.17 | -0.11 | -0.13 | 0.06 | 0.20 | -0.07 | -0.19 | 0.00 | ND | ND |
| 7 | **0.70** | -0.08 | 0.03 | 0.05 | 0.08 | -0.23 | 0.00 | -0.12 | -0.17 | 0.19 | ND | ND |
| 8 | **0.70** | -0.03 | -0.06 | 0.05 | 0.09 | -0.23 | -0.12 | 0.10 | -0.02 | 0.17 | ND | ND |
| 9 | **0.83** | -0.02 | 0.00 | -0.12 | -0.16 | 0.07 | -0.02 | -0.10 | 0.05 | -0.16 | ND | ND |
| 10 | **0.81** | 0.00 | -0.05 | -0.13 | -0.09 | 0.05 | -0.04 | -0.02 | 0.15 | -0.24 | ND | ND |
| 11 | 0.26 | -0.15 | 0.01 | -0.12 | -0.01 | **0.51** | 0.06 | -0.25 | 0.10 | 0.42 | NW | NW |
| 12 | 0.18 | -0.20 | -0.12 | -0.08 | 0.11 | **0.67** | -0.22 | -0.13 | 0.05 | 0.24 | NW | NW |
| 13 | -0.21 | -0.04 | 0.05 | -0.03 | -0.04 | **0.82** | 0.07 | -0.03 | -0.06 | -0.08 | NW | NW |
| 14 | -0.23 | -0.06 | 0.18 | 0.09 | -0.05 | **0.74** | 0.07 | 0.07 | -0.16 | 0.12 | NW | NW |
| 15 | -0.08 | -0.06 | -0.04 | 0.03 | -0.10 | 0.20 | -0.06 | -0.12 | -0.10 | **0.75** | NW | Factor 10 |
| 16 | -0.05 | 0.17 | 0.26 | **0.49** | 0.01 | 0.24 | -0.01 | 0.05 | -0.16 | -0.29 | AR | SR |
| 17 | -0.08 | 0.11 | **0.62** | 0.09 | 0.14 | 0.07 | 0.31 | 0.25 | 0.03 | -0.04 | AR | AR |
| 18 | -0.20 | 0.19 | **0.44** | -0.13 | -0.01 | -0.24 | 0.40 | 0.00 | -0.10 | 0.16 | AR | AR |
| 19 | -0.07 | 0.04 | **0.82** | 0.05 | 0.08 | -0.03 | 0.04 | -0.07 | 0.08 | 0.05 | AR | AR |
| 20 | -0.01 | 0.05 | **0.72** | 0.07 | -0.01 | 0.01 | 0.18 | -0.07 | 0.10 | -0.02 | AR | AR |
| 21 | -0.07 | 0.18 | **0.63** | 0.21 | 0.16 | 0.14 | -0.03 | 0.16 | 0.06 | -0.08 | AR | AR |
| 22 | -0.01 | 0.21 | **0.59** | 0.30 | 0.14 | 0.12 | -0.15 | 0.15 | 0.23 | -0.18 | AR | AR |
| 23 | 0.05 | **0.62** | 0.21 | -0.03 | 0.00 | 0.00 | 0.07 | 0.28 | 0.20 | 0.13 | EA | EA |
| 24 | 0.01 | **0.63** | 0.09 | 0.04 | 0.04 | -0.17 | 0.10 | 0.32 | -0.04 | 0.02 | EA | EA |
| 25 | -0.04 | **0.77** | 0.08 | 0.02 | 0.25 | -0.02 | 0.20 | -0.02 | 0.07 | -0.11 | EA | EA |
| 26 | -0.06 | **0.68** | 0.03 | 0.29 | 0.09 | -0.11 | 0.10 | -0.02 | 0.17 | -0.06 | EA | EA |
| 27 | 0.04 | **0.82** | 0.15 | 0.15 | 0.15 | -0.05 | 0.09 | -0.06 | 0.01 | -0.06 | EA | EA |
| 28 | -0.04 | 0.08 | 0.17 | **0.49** | 0.15 | 0.17 | 0.02 | -0.44 | 0.17 | 0.09 | SR | SR |
| 29 | 0.01 | 0.29 | 0.22 | **0.56** | 0.35 | -0.04 | 0.10 | -0.15 | 0.14 | -0.14 | SR | SR |
| 30 | -0.10 | 0.02 | 0.01 | **0.79** | 0.12 | -0.17 | 0.13 | 0.07 | 0.08 | 0.06 | SR | SR |
| 31 | -0.09 | 0.10 | 0.14 | **0.79** | 0.00 | -0.01 | 0.19 | 0.11 | -0.01 | 0.07 | SR | SR |
| 32 | 0.08 | 0.28 | 0.17 | 0.29 | 0.18 | -0.07 | **0.58** | 0.28 | -0.03 | -0.10 | BL | BL |
| 33 | 0.00 | 0.28 | 0.16 | 0.31 | 0.22 | 0.07 | **0.65** | -0.02 | 0.07 | -0.12 | BL | BL |
| 34 | -0.01 | 0.15 | 0.13 | 0.13 | 0.33 | 0.06 | **0.64** | 0.09 | 0.17 | 0.03 | BL | BL |
| 35 | -0.11 | 0.17 | 0.17 | 0.07 | **0.78** | -0.03 | 0.18 | -0.09 | 0.02 | 0.09 | T | T |
| 36 | -0.06 | 0.13 | 0.09 | 0.11 | **0.86** | 0.02 | 0.07 | 0.05 | 0.04 | -0.01 | T | T |
| 37 | 0.05 | 0.12 | 0.05 | 0.11 | **0.78** | -0.01 | 0.18 | 0.20 | -0.03 | -0.16 | T | T |

*Note*. N, Noticing; ND, Not-Distracting; NW, Not-Worrying; A, Attention Regulation; E, Emotional Awareness; S, Self-Regulation; B, Body Listening; T, Trusting.

**Supplementary eTable 3.**

**Items, communality, and exploratory factor analysis (EFA) loadings of the Chinese version of the MAIA.**

| Items and factors | | Factor loadings | Commun-alities |  |
| --- | --- | --- | --- | --- |
|  |  |  |  |  |
| Not-Distracting (Cronbach’s α = 0.803) | | | |  |
| 5 | I ignore physical tension or discomfort until they become more severe.  我会忽视身体的紧张或不适感，直到症状愈发严重。 | 0.47 | 0.36 |  |
| 6 | I distract myself from sensations of discomfort.  我会分散自己对不舒服感觉的注意力。 | 0.71 | 0.65 |  |
| 7 | When I feel pain or discomfort, I try to power through it.  当我感到疼痛或不舒服时，我会尝试挺过去。 | 0.73 | 0.58 |  |
| 8 | I try to ignore pain.  我会尝试忽略疼痛。 | 0.72 | 0.57 |  |
| 9 | I push feelings of discomfort away by focusing on something.  我会通过把注意力专注于其他事情上来消除不舒服的感觉。 | 0.81 | 0.72 |  |
| 10 | When I feel unpleasant body sensations, I occupy myself with something else, so I don’t have to feel them.  当我感觉身体不舒服的时候，我会让自己专注于其他事情，这样我就可以不必感受这些感觉了。 | 0.78 | 0.64 |  |
|  |  |  |  |  |
| Not-Worrying (Cronbach’s α = 0.656) | | | |  |
| 11 | When I feel physical pain, I become upset.  当我感受到身体疼痛的时候，我会变得心烦。 | 0.62 | 0.49 |  |
| 12 | I start to worry that something is wrong if I feel any discomfort.  如果我感到任何不适，我会开始担心我的身体是不是出了什么问题。 | 0.72 | 0.67 |  |
|  |  |  |  |  |
| 13 | I can notice an unpleasant body sensation without worrying about it.  我能注意到身体不舒服的感觉，但不会担心它。 | 0.79 | 0.72 |  |
| 14 | I can stay calm and not worry when I have feelings of discomfort or pain.  当我有不舒服或疼痛的感觉时，我能保持冷静且不为之担心。 | 0.73 | 0.66 |  |
| Attention Regulation (Cronbach’s α = 0.822) | | | |  |
| 17 | I can maintain awareness of my inner bodily sensations even when there is a lot going on around me.  即使身边有很多事情正在发生，我依然可以维持对内部身体感觉的觉察。 | 0.62 | 0.58 |  |
|  |  |  |  |  |
| 18 | When I am in conversation with someone, I can pay attention to my posture.  当与他人交谈时，我会注意自己的姿势。 | 0.39 | 0.40 |  |
| 19 | I can return awareness to my body if I am distracted.  即使注意力被分散了，我也能将意识重新拉回到自己身体。 | 0.81 | 0.68 |  |
| 20 | I can refocus my attention from thinking to sensing my body.  我可以在思考的时候把注意力转回到感知我的身体上。 | 0.72 | 0.57 |  |
| 21 | I can maintain awareness of my whole body even when a part of me is in pain or discomfort.  即使当身体的某个部分感到疼痛或不舒服时，我依然能保持对整个身体的觉察。 | 0.65 | 0.54 |  |
|  |  |  |  |  |
| 22 | I am able to consciously focus on my body as a whole.  我能有意识地把注意力集中在我整个身体上。 | 0.64 | 0.60 |  |
| Emotional Awareness (Cronbach’s α = 0.817) | | | |  |
| 23 | I notice how my body changes when I am angry.  当我感到生气时，我能察觉到身体发生了怎样的变化。 | 0.66 | 0.51 |  |
| 24 | When something is wrong in my life I can feel it in my body.  当生活中出现问题时，我能在身体中感受到它带来的影响。 | 0.65 | 0.51 |  |
| 25 | I notice that my body feels different after a peaceful experience.  在一段令人愉悦和舒服的经历后，我注意到我的身体感觉会有所不同。 | 0.76 | 0.68 |  |
|  |  |  |  |  |
| 26 | I notice that my breathing becomes free and easy when I feel  comfortable.  当我觉得舒服的时候，我注意到我的呼吸变得自由而轻松。 | 0.68 | 0.60 |  |
| 27 | I notice how my body changes when I feel happy/joyful.  当我感到快乐时，我能察觉到身体发生了怎样的变化。 | 0.80 | 0.72 |  |
| Self-Regulation (Cronbach’s α = 0.741) | | | |  |
| 28 | When I feel overwhelmed, I can find a calm place inside.  当我感到不堪重负的时候，我依然能在内心找到一份安宁。 | 0.61 | 0.49 |  |
| 29 | When I bring awareness to my body, I feel a sense of calm.  当我有意识地感知我的身体时，我会有一种平静感。 | 0.61 | 0.61 |  |
| 30 | I can use my breath to reduce tension.  我可以通过呼吸来减少紧张感。 | 0.78 | 0.69 |  |
| 31 | When I am caught up in thoughts, I can calm my mind by focusing on my body/breathing.  当我纠结于某件事情时，我能通过把注意力集中在我的身体或呼吸上来让自己冷静。 | 0.76 | 0.68 |  |
|  |  |  |  |  |
| Body Listening (Cronbach’s α = 0.765) | | | |  |
| 32 | I listen for information from my body about my emotional state.  我会留意来自身体中的有关情绪状态的信息。 | 0.67 | 0.67 |  |
| 33 | When I am upset, I take time to explore how my body feels.  当我心烦的时候，我会花时间去探知我身体的感受。 | 0.63 | 0.65 |  |
| 34 | I listen to my body to inform me about what to do.  我会听从身体来告诉我该做什么。 | 0.61 | 0.56 |  |
| Trusting (Cronbach’s α = 0.838) | | | |  |
| 35 | I am at home in my body.  我在自己身体里感到很放松和自在。 | 0.79 | 0.72 |  |
| 36 | I feel my body is a safe place.  我觉得我的身体是一个安全的地方。 | 0.86 | 0.79 |  |
| 37 | I trust my body sensations.  我相信自己身体的感觉。 | 0.77 | 0.68 |  |
